# Self-normal and biorthogonal dynamical quantum phase transitions in non-Hermitian quantum walks

**DOI:** 10.1038/s41377-025-01919-6

**Published:** 2025-07-26

**Authors:** Haiting Zhang, Kunkun Wang, Lei Xiao, Peng Xue

**Affiliations:** 1 Beijing Computational Science Research Center, Beijing, 100193 China; 2 School of Physics and Optoelectronic Engineering, Anhui University, Hefei, 230601 China; 3 Key Laboratory of Quantum Materials and Devices of Ministry of Education, School of Physics, Southeast University, Nanjing, 211189 China

**Keywords:** Quantum optics, Single photons and quantum effects

## Abstract

Dynamical quantum phase transitions (DQPTs), characterized by non-analytic behavior in rate function and abrupt changes in dynamic topological order parameters (DTOPs) over time, have garnered enormous attention in recent decades. However, in non-Hermitian systems, the special biorthogonality of the bases makes the definition of DQPTs complex. In this work, we delve into the comprehensive investigation of self-normal DQPTs (originally used in Hermitian systems) to compare them with their biorthogonal counterpart, within the context of non-Hermitian quantum walks (QWs). We present a detailed analysis of the behaviors of Loschmidt rate functions and DTOPs under these two distinct theoretical approaches. While both self-normal and biorthogonal methods can be used to detect DQPTs in quench dynamics between different topological phases, we theoretically present their differences in the definition of critical momenta and critical times by analyzing the Fisher zeros and fixed points. Finally, we present an experiment that observes both types of DQPTs using one-dimensional discrete-time QWs with single photons.

## Abstract (web summary)

The article performs a detailed analysis of self-normal and biorthogonal dynamical quantum phase transitions, presenting characteristics of their physical quantities both theoretically and experimentally.

## Graphical abstract

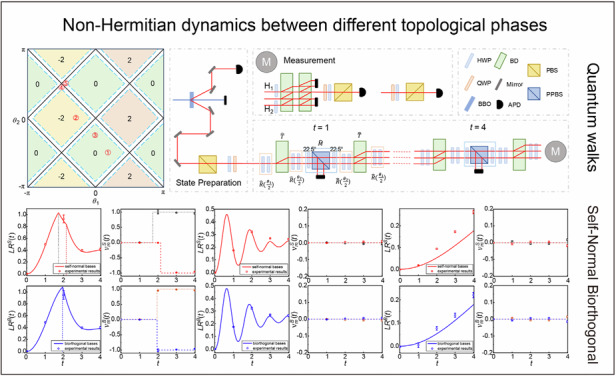

## Introduction

Equilibrium phase transitions^1,2^ are typically characterized by non-analyticities in free-energy density, marked by the zeros of the partition function, which are also known as Fisher zeros in the complex temperature or magnetic field plane. When this idea is generalized to nonequilibrium systems, the dynamical free-energy density becomes singular at certain critical times, indicating the dynamical quantum phase transitions (DQPTs)^3,4^. Over the past few decades, DQPTs have become a vast interesting topic and have attracted significant attention. The realm of DQPTs is extended from free fermion models^5^ to interacting systems^6,7^ as well as bosonic systems^8,9^. In the context of Floquet driving^10^, Fisher zeros exhibit intriguing profiles^11^. DQPTs are extensively studied in the context of quantum information theoretic measures, namely decoherence^12^. In particular, DQPTs have been experimentally observed in various platforms like trapped-ion^13,14^, nuclear magnetic resonance^15^, Rydberg atoms^16^, ultracold atoms^17^, superconducting qubits^18^, and photonic lattices^19,20^.

Previously, most studies on DQPTs have primarily focused on closed Hermitian quantum systems governed by unitary time evolution. At the critical time, DQPTs occur when the time-evolved state $| \varPsi (t)\left.\right\rangle$ becomes orthogonal to the initial state $| \varPsi (0)\left.\right\rangle$, leading to the non-analytical behavior in Loschmidt rate function^21,22^. Besides, analogous to the order parameter in conventional phase transitions, dynamic topological order parameters (DTOPs)^23,24^ serve as physical observables for DQPTs. These parameters, derived from the Pancharatnam geometric phase^25,26^, provide insights into the topological nature of DQPTs.

Recently, DQPTs in non-Hermitian systems with gain and loss^27,28^, and therefore governed by non-unitary evolution, have been extensively studied both theoretically^23,29,30^ and experimentally^20,31^. However, defining the Loschmidt echo or DTOPs poses significant challenges due to the special biorthogonality between the left and right eigenstates of the non-Hermitian Hamiltonian. Early efforts employed self-normalized bases^32^ to describe non-Hermitian DQPTs, often enforcing a normalization factor^33^ while ignoring biorthogonality. Very recently, it has been shown that DQPTs in non-Hermitian systems require a novel biorthogonal theoretical approach^34^ with biorthogonal bases^32^ and associated states. Since these two kinds of DQPTs have different physical origins, it is a natural question to ask: How can their differences be revealed both theoretically and experimentally?

In this work, we elucidate the concept of self-normal DQPTs under the framework of Hermitian quantum mechanics and biorthogonal DQPTs under the framework of non-Hermitian quantum mechanics. Specifically, we perform a detailed analysis of these two types of DQPTs, presenting their characteristics of the Loschmidt rate function, DTOPs, and Fisher zeros in momentum space. Moreover, we carry out our study in one-dimensional quantum walks (QWs), which incorporate non-Hermiticity and exhibit distinct topological phases in PT-symmetry-broken regions. We investigate the dynamics following a sudden quench of the controlling parameters, i.e., coin parameters *θ*_1_, *θ*_2_, in QWs. Through numerical analysis of various physical observables, we demonstrate that these two types of DQPTs exhibit distinct behaviors in one-dimensional non-Hermitian QWs. Furthermore, we reveal the differences in critical momenta and critical times between these two types of DQPTs within PT-symmetry-unbroken regions. By contrast, if either the post-quench Hamiltonian is in the PT-symmetry-broken regions, the appearance of these two types of DQPTs is not found.

To support the above theoretical results, we perform an experiment that observes different types of DQPTs using one-dimensional discrete-time QWs of single photons. In particular, we simulate non-unitary quench dynamics of PT-symmetric systems by introducing controllable photon loss. By reconstructing the time-evolved states through measurements in position space, we evaluate both the self-normal and biorthogonal Loschmidt rate functions, as well as the corresponding DTOPs, to characterize these two types of DQPTs.

## Results

### Self-normal and biorthogonal bases

Consider a split-step non-Hermitian QW governed by the operator $\hat{U}$, whose effective Hamiltonian is $\hat{H}$. For the non-Hermitian QW system of ${\hat{U}}_{k}$ in the momentum space, the eigenvalue equations are given by

1 $$\begin{array}{r}{\hat{U}}_{k}| {\varphi }_{k,\omega }^{R}\left.\right\rangle ={\mu }_{k,\omega }| {\varphi }_{k,\omega }^{R}\left.\right\rangle ,\left\langle \right.{\varphi }_{k,\omega }^{R}| {\hat{U}}_{k}^{\dagger }=\left\langle \right.{\varphi }_{k,\omega }^{R}| {\mu }_{k,\omega }^{* }\\ {\hat{U}}_{k}^{\dagger }| {\varphi }_{k,\omega }^{L}\left.\right\rangle ={\mu }_{k,\omega }^{* }| {\varphi }_{k,\omega }^{L}\left.\right\rangle ,\left\langle \right.{\varphi }_{k,\omega }^{L}| {\hat{U}}_{k}=\left\langle \right.{\varphi }_{k,\omega }^{L}| {\mu }_{k,\omega }\end{array}$$

where *μ*_*k*,*ω*_ are the eigenvalues in *k* space, $| {\varphi }_{k,\omega }^{R}\left.\right\rangle$ and $| {\varphi }_{k,\omega }^{L}\left.\right\rangle$ are the right and left eigenvectors, respectively. Moreover, *ω* =± denotes two energy bands for two-level systems. Thus, based on the above equations, we can define two separate classes of bases. Similarly to its Hermitian counterpart, we establish self-normal bases using only the right eigenstates as follows

2 $$| {\psi }_{k,\omega }^{R}\left.\right\rangle =\frac{| {\varphi }_{k,\omega }^{R}\left.\right\rangle }{\sqrt{\langle {\varphi }_{k,\omega }^{R}| {\varphi }_{k,\omega }^{R}\rangle }}$$

However, the self-normal bases are non-orthonormal, i.e., ${\sum }_{\omega }| {\psi }_{k,\omega }^{R}\left.\right\rangle \left\langle \right.{\psi }_{k,\omega }^{R}| \,\ne\, I$, due to the non-Hermiticity of systems although each self-normal basis can be normalized $\langle {\psi }_{k,\omega }^{R}| {\psi }_{k,{\omega }^{{\prime} }}^{R}\rangle ={\delta }_{\omega ,{\omega }^{{\prime} }}$, independently. Alternatively, we can define biorthogonal bases by combining both left eigenstates and right eigenstates as follows

3 $$\begin{array}{r}| {\tilde{\psi }}_{k,\omega }^{L}\left.\right\rangle =\frac{| {\varphi }_{k,\omega }^{L}\left.\right\rangle }{\sqrt{\langle {\varphi }_{k,\omega }^{R}| {\varphi }_{k,\omega }^{L}\rangle }},| {\tilde{\psi }}_{k,\omega }^{R}\left.\right\rangle =\frac{| {\varphi }_{k,\omega }^{R}\left.\right\rangle }{\sqrt{\langle {\varphi }_{k,\omega }^{L}| {\varphi }_{k,\omega }^{R}\rangle }}\end{array}$$

Here the biorthogonal bases satisfy the completeness relation ${\sum }_{\omega }| {\tilde{\psi }}_{k,\omega }^{L}\left.\right\rangle \left\langle \right.{\tilde{\psi }}_{k,\omega }^{R}| =I$ and the biorthonornal relation $\langle {\tilde{\psi }}_{k,\omega }^{L}| {\tilde{\psi }}_{k,{\omega }^{{\prime} }}^{R}\rangle ={\delta }_{\omega ,{\omega }^{{\prime} }}$. Relying on these two frameworks, two major approaches for investigating the DQPTs of non-Hermitian systems have been derived: self-normal DQPTs and biorthogonal DQPTs.

### Self-normal dynamical quantum phase transitions

At *t* = 0, the system undergoes a sudden quench switched from ${\hat{H}}^{i}$ to ${\hat{H}}^{f}$, which is governed by ${\hat{U}}^{f}$. We initially prepare the state in the ground state $| {\varPsi }_{k}^{R}(0)\left.\right\rangle =| {\psi }_{k,-}^{R,i}\left.\right\rangle$ of the initial effective Hamiltonian ${\hat{H}}_{k}^{i}$. The translational symmetry allows us to treat the dynamics of each state $| {\psi }_{k,-}^{R,i}\left.\right\rangle$ in different quasimomentum *k*-sectors. Afterwards, under the repeated operation of ${\hat{U}}_{k}^{f}$, the photon at the *t*th step evolves to $| {\varPsi }_{k}^{R}(t)\left.\right\rangle ={({\hat{U}}_{k}^{f})}^{t}| {\varPsi }_{k}^{R}(0)\left.\right\rangle ={e}^{-i{\hat{H}}_{k}^{f}t}| {\psi }_{k,-}^{R,i}\left.\right\rangle$ in terms of individual momentum modes.

The overlap between $| {\varPsi }_{k}^{R}(0)\left.\right\rangle$ and $| {\varPsi }_{k}^{R}(t)\left.\right\rangle$ is characterized by the self-normal Loschmidt echo (see Sec. S1A of the Supplementary Material for more details)

4 $$L{E}^{S}(t)=\prod _{k}\frac{| \langle {\varPsi }_{k}^{R}(0)| {\varPsi }_{k}^{R}(t)\rangle {| }^{2}}{\langle {\varPsi }_{k}^{R}(t)| {\varPsi }_{k}^{R}(t)\rangle }$$

where $L{A}_{k}^{S}(t)=\langle {\varPsi }_{k}^{R}(0)| {\varPsi }_{k}^{R}(t)\rangle$ is the self-normal return amplitude and $\langle {\varPsi }_{k}^{R}(t)| {\varPsi }_{k}^{R}(t)\rangle$ is the enforced normalized factor to avoid the negative value of the self-normal Loschmidt rate. When the Loschmidt echo in Eq. (4) becomes zero, the self-normal DQPT occurs, corresponding to non-analyticities in the self-normal Loschmidt rate function. In the thermodynamic limit, the self-normal Loschmidt rate function is given by

5 $$L{R}^{S}(t)=-\frac{1}{2\pi }\mathop{\int}\nolimits_{0}^{2\pi }dk\ln [L{E}_{k}^{S}(t)]$$

Besides, a self-normal DQPT is expected when a line of Fisher zeros *z*_*n*,*k*_ (see Materials and Methods) crosses the imaginary time axis at the momentum $k={k}_{c}^{S}$, yielding a critical time ${t}_{n,c}^{S}=-i{z}_{n,{k}_{c}^{S}}$. Here *n* denotes an integer.

On the other hand, the self-normal DQPTs are characterized by each self-normal DTOP of ${\nu }_{m}^{S}(t)$, which is defined through the Pancharatnam geometric phase ${\phi }_{k}^{S,G}(t)$ as

6 $${\nu }_{m}^{S}(t)=\frac{1}{2\pi }\mathop{\int}\nolimits_{{k}_{m}^{S}}^{{k}_{m+1}^{S}}dk\left[{\partial }_{k}{\phi }_{k}^{{\rm{S,G}}}(t)\right]$$

Here ${k}_{m}^{S}\,(m=1,2,\ldots )$ are fixed points^31,35^ of the self-normal DQPTs. While fixed points necessarily exist when ${\hat{U}}^{i}$ and ${\hat{U}}^{f}$ have different winding numbers, ${k}_{c}^{S}$ exists between adjacent fixed points and leads to DQPTs. As a result, ${\nu }_{m}^{S}(t)$ exhibits abrupt jumps at the self-normal DQPTs, which are associated with ${k}_{c}^{S}\in ({k}_{m}^{S},{k}_{m+1}^{S})$. The self-normal Pancharatnam geometric phase is given by ${\phi }_{k}^{S,G}(t)={\phi }_{k}^{S}(t)-{\phi }_{k}^{S,dyn}(t)$ with the self-normal total phase ${\phi }_{k}^{S}(t)$ and the self-normal dynamical phase ${\phi }_{k}^{S,dyn}(t)$^36^ (Sec. S1A of the Supplementary Material). For clarity, we summarize key self-normal physical quantities in Table S1 of the Supplementary Material.

### Biorthogonal dynamical quantum phase transitions

In terms of biorthogonal DQPTs, we prepare the initial state in the biorthogonal ground state $| {\tilde{\varPsi }}_{k}^{R}(0)\left.\right\rangle =| {\tilde{\psi }}_{k,-}^{R,i}\left.\right\rangle$. Under the repeated operation of ${\hat{U}}_{k}^{f}$, the time evolution state is given by the Schrödinger equation $| {\tilde{\varPsi }}_{k}^{R}(t)\left.\right\rangle ={({\hat{U}}_{k}^{f})}^{t}| {\tilde{\varPsi }}_{k}^{R}(0)\left.\right\rangle ={e}^{-i{\hat{H}}_{k}^{f}t}| {\tilde{\psi }}_{k,-}^{R,i}\left.\right\rangle$ in momentum space. However, the direct generalization $| {\tilde{\varPsi }}_{k}^{L}(t)\left.\right\rangle$ may lead to unreasonable complex probabilities. To reconcile the negative transition probability, the associated state is introduced. For the evolved state $| {\tilde{\varPsi }}_{k}^{R}(t)\left.\right\rangle$, its associated state $| {\tilde{\varPsi }}_{k}^{L}(t)\left.\right\rangle$ is defined according to the following relation^32^

7 $$\begin{array}{c}| {\tilde{\varPsi }}_{k}^{R}(t)\left.\right\rangle ={c}_{k,+}(t)| {\tilde{\psi }}_{k,+}^{R,i}\left.\right\rangle +{c}_{k,-}(t)| {\tilde{\psi }}_{k,-}^{R,i}\left.\right\rangle \\ \Updownarrow \\ | {\tilde{\varPsi }}_{k}^{L}(t)\left.\right\rangle ={c}_{k,+}(t)| {\tilde{\psi }}_{k,+}^{L,i}\left.\right\rangle +{c}_{k,-}(t)| {\tilde{\psi }}_{k,-}^{L,i}\left.\right\rangle \end{array}$$

where ${c}_{k,+}(t)=\langle {\tilde{\psi }}_{k,+}^{L,i}| {\tilde{\varPsi }}_{k}^{R}(t)\rangle$ and ${c}_{k,-}(t)=\langle {\tilde{\psi }}_{k,-}^{L,i}| {\tilde{\varPsi }}_{k}^{R}(t)\rangle$ (Sec. S1B of the Supplementary Material). The dual evolved state $\left\langle \right.{\tilde{\varPsi }}_{k}^{R}(t)|$ and its associated state $\left\langle \right.{\tilde{\varPsi }}_{k}^{L}(t)|$ are defined as follows:

8 $$\begin{array}{rcl}\left\langle \right.{\tilde{\varPsi }}_{k}^{R}(t)| &=&\left\langle \right.{\tilde{\psi }}_{k,+}^{R,i}| {c}_{k,+}^{* }(t)+\left\langle \right.{\tilde{\psi }}_{k,-}^{R,i}| {c}_{k,-}^{* }(t)\\ \left\langle \right.{\tilde{\varPsi }}_{k}^{L}(t)| &=&\left\langle \right.{\tilde{\psi }}_{k,+}^{L,i}| {c}_{k,+}^{* }(t)+\left\langle \right.{\tilde{\psi }}_{k,-}^{L,i}| {c}_{k,-}^{* }(t)\end{array}$$

Through Eq. (7) and Eq. (8), the biorthogonal Loschmidt echo is given by $L{E}^{B}(t)={\prod }_{k}L{E}_{k}^{B}(t)$ with (details are given in Sec. S1B of the Supplementary Material)

9 $$L{E}_{k}^{B}(t)=\frac{\langle {\tilde{\varPsi }}_{k}^{L}(0)| {\tilde{\varPsi }}_{k}^{R}(t)\rangle \langle {\tilde{\varPsi }}_{k}^{L}(t)| {\tilde{\varPsi }}_{k}^{R}(0)\rangle }{\langle {\tilde{\varPsi }}_{k}^{L}(t)| {\tilde{\varPsi }}_{k}^{R}(t)\rangle }$$

In the thermodynamic limit, the biorthogonal rate function of the Loschmidt echo is given by

10 $$L{R}^{B}(t)=-\frac{1}{2\pi }\mathop{\int}\nolimits_{0}^{2\pi }dk\ln \left[L{E}_{k}^{B}(t)\right]$$

The non-analyticities in the biorthogonal rate function appear when a line of biorthogonal Fisher zeros (see Materials and Methods) crosses the imaginary time axis at momentum $k={k}_{c}^{B}$, resulting in a critical time ${t}_{n,c}^{B}=-i{z}_{n,{k}_{c}^{B}}$, satisfying Re$[{z}_{n,{k}_{c}^{B}}]=0$. Here *n* denotes the integer numbers.

Similar to the DTOP in Hermitian systems, the biorthogonal DTOP of ${\nu }_{m}^{B}(t)$ is defined as

11 $${\nu }_{m}^{B}(t)=\frac{1}{2\pi }\mathop{\int}\nolimits_{{k}_{m}^{B}}^{{k}_{m+1}^{B}}dk\left[{\partial }_{k}{\phi }_{k}^{B,G}(t)\right]$$

Here ${k}_{m}^{B}\,(m=1,2,\ldots )$ are fixed points of the biorthogonal DQPTs. As a result, ${\nu }_{m}^{B}(t)$ exhibits abrupt jumps at the biorthogonal DQPTs associated with ${k}_{c}^{B}\in ({k}_{m}^{B},{k}_{m+1}^{B})$. The biorthogonal geometric phase of the return amplitude is given by ${\phi }_{k}^{B,G}(t)={\phi }_{k}^{B}(t)-{\phi }_{k}^{B,dyn}(t)$ with the biorthogonal total phase ${\phi }_{k}^{B}(t)$ and the biorthogonal dynamical phase ${\phi }_{k}^{B,dyn}(t)$ (Sec. S1B of the Supplementary Material). For clarity, we summarize key biorthogonal physical quantities in Table S1 of the Supplementary Material.

### Split-step quantum walks

We investigate DQPTs under a quench dynamics using split-step QWs^20,31,37–41^ on one-dimensional homogeneous lattice *L* (*L* ∈ *Z*). The dynamics is governed by the non-unitary topological Floquet operator

12 $$\hat{U}=\gamma \hat{R}\left(\frac{{\theta }_{1}}{2}\right)\hat{T}\hat{R}\left(\frac{{\theta }_{2}}{2}\right)\hat{M}\hat{R}\left(\frac{{\theta }_{2}}{2}\right)\hat{T}\hat{R}\left(\frac{{\theta }_{1}}{2}\right)$$

where $\gamma ={(1-l)}^{-\frac{1}{4}}$ is a tunable gain/loss parameter. The coin operator $\hat{R}(\theta )={\sum }_{x}| x\left.\right\rangle \left\langle \right.x| \otimes {e}^{-i\theta {\hat{\sigma }}_{y}}$ rotates coin states by *θ* around the *y*-axis, and the translation operator $\hat{T}={\sum }_{x}| x+1\left.\right\rangle \langle x| \otimes | H\rangle \langle H| +| x-1\rangle \langle x| \otimes | V\rangle \left\langle \right.V|$ denotes a translation to the right (left) by one lattice site depending on the coin state $\left\vert H\right\rangle$ ($\left\vert V\right\rangle$). Here ${\hat{\sigma }}_{y}=i(-| H\left.\right\rangle \langle V| +| V\rangle \left\langle \right.H| )$ is one of the Pauli operators. The loss operator $\hat{M}={\sum }_{x}| x\left.\right\rangle \left\langle \right.x| \otimes (| +\left.\right\rangle \langle +| +\sqrt{1-l}| -\rangle \left\langle \right.-| )$ ($| \pm \left.\right\rangle =(| H\left.\right\rangle \pm | V\left.\right\rangle )/\sqrt{2}$) enforces mode-selective photon losses with loss parameter *l* ∈ (0, 1]. The operator $\hat{U}$ exists PT-symmetry and satisfies the relation $\hat{P}\hat{T}\hat{U}{(\hat{P}\hat{T})}^{-1}={\hat{U}}^{-1}$, where the symmetry operator is given by $\hat{P}\hat{T}={\sum }_{x}| -x\left.\right\rangle \left\langle \right.x| \otimes \hat{{\sigma }_{z}}\hat{\kappa }$ with the complex conjugation operator $\hat{\kappa }$.

The effective Hamiltonian is obtained from $\hat{U}={e}^{-i\hat{H}}$. In the quasimomentum space, it can be presented as

13 $${\hat{H}}_{k}=\int\,dk{E}_{k}\hat{n}\cdot \hat{\sigma }\otimes | k\left.\right\rangle \left\langle \right.k|$$

where $\hat{\sigma }=({\hat{\sigma }}_{x},{\hat{\sigma }}_{y},{\hat{\sigma }}_{z})$ is the Pauli matrix vector, and the unit vector $\hat{n}=({\hat{n}}_{x},{\hat{n}}_{y},{\hat{n}}_{z})$ determines the spin eigenstate at each quasienergy ±*E*_*k*_. It is straightforward to check that $\hat{H}$ possesses the chiral symmetry of the form ${\hat{\sigma }}_{x}\hat{H}{\hat{\sigma }}_{x}=-\hat{H}$. From this perspective, the QW can exhibit interesting topological properties, with winding numbers defined as the count of times $\hat{n}$ winds around the *x*-axis as *k* varies through the first Brillouin zone^42^.

Due to the periodic implementation of $\hat{U}$, the quasienergy acts as an accumulated phase for the eigenstate after each step and has a periodicity of 2*π*^43–45^. To begin with, upon applying the Fourier transform, the Floquet operator $\hat{U}$ in each *k*-sector can be formulated as

14 $$\begin{array}{lll}{\hat{U}}_{k}&=&{n}_{0}{\hat{\sigma }}_{0}-i{n}_{1}{\hat{\sigma }}_{x}-i{n}_{2}{\hat{\sigma }}_{y}-i{n}_{3}{\hat{\sigma }}_{z}\\ {n}_{0}&=&\alpha (\cos (2k)\cos {\theta }_{1}\cos {\theta }_{2}-\sin {\theta }_{1}\sin {\theta }_{2})\\ {n}_{1}&=&i\beta \\ {n}_{2}&=&\alpha (\cos (2k)\cos {\theta }_{2}\sin {\theta }_{1}-\cos {\theta }_{1}\sin {\theta }_{2})\\ {n}_{3}&=&-\alpha \sin (2k)\cos {\theta }_{2}\end{array}$$

where $\alpha =\gamma (1+\sqrt{1-l})/2$, $\beta =\gamma (1-\sqrt{1-l})/2$, and $\gamma ={(1-l)}^{-\frac{1}{4}}$. The eigenvalues of ${\hat{U}}_{k}$ are ${\mu }_{k,\pm }={n}_{0}\mp i\sqrt{1-{n}_{0}^{2}}$ and the quasienergies are ±*E*_*k*_ = $i\ln ({\mu }_{k,\pm })$. When ${n}_{0}^{2} < 1$ for all *k*, the system is in the PT-symmetry-unbroken region and the quasienergy is real. When ${n}_{0}^{2}\ge 1$ for some *k*, the system is in the PT-symmetry-broken region and the quasienergy is imaginary. Therefore, we can generalize the definition of winding number^37^

15 $$\nu =-\frac{1}{2\pi }\sum _{\omega =\pm }\mathop{\int}\nolimits_{0}^{2\pi }{\rm{d}}k\frac{\langle {\tilde{\psi }}_{k,\omega }^{L}| i{\partial }_{k}| {\tilde{\psi }}_{k,\omega }^{R}\rangle }{\langle {\tilde{\psi }}_{k,\omega }^{L}| {\tilde{\psi }}_{k,\omega }^{R}\rangle }$$

Here the right (left) eigenvector is defined as Eq. (3) in each *k*-sector. Winding numbers are also labeled in the phase diagram to characterize different topological phases.

**Fig. 1:**
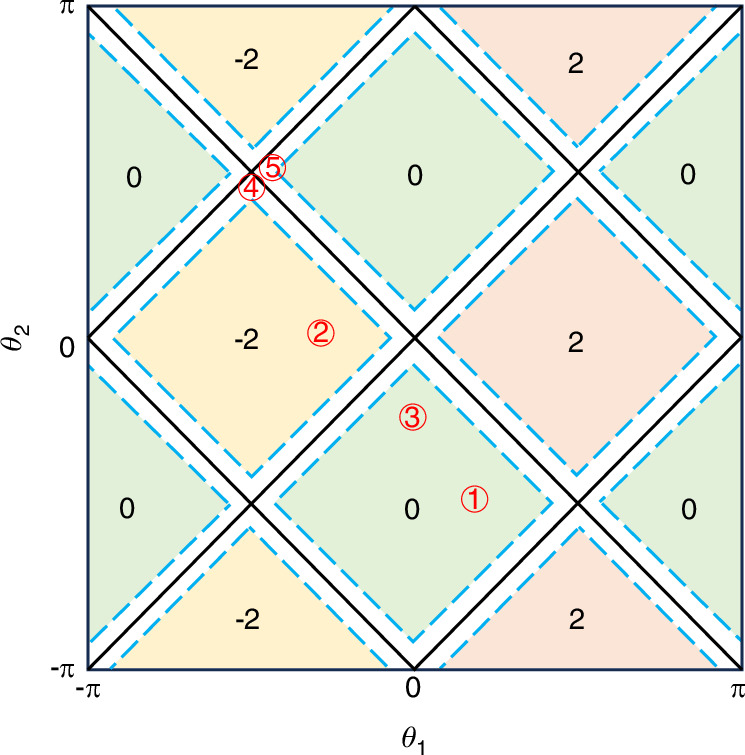
Phase diagram. Phase diagram of a non-unitary QW in Eq. (12). Topological phases are distinguished by the winding number *ν* as a function of the coin parameters *θ*_1_ and *θ*_2_. The black lines denote the topological phase boundaries, separating the parameter space into three distinct phases (yellow, magenta and green). The blue dashed lines represent the boundaries between PT-symmetry-unbroken and PT-symmetry-broken regions. When Im[*E*_*k*_] ≠ 0, it indicates that the PT-symmetry-broken regions are located between the blue dashed lines near the topological phase boundaries. ① denotes the coin parameters of the initial Floquet operators ${\hat{U}}^{i}$. ②-⑤ indicate the coin parameters of different final Floquet operators ${\hat{U}}^{f}$

As illustrated in Fig. 1, we display the topological phase diagram with distinct winding numbers by varying coin parameters (*θ*_1_, *θ*_2_). In yellow and magenta regions, which correspond to the winding number *ν* =− 2 and *ν* = 2, respectively, indicating the system is topologically nontrivial. However, for green regions, the system is topologically trivial with *ν* = 0. The boundaries between the PT-symmetry-unbroken and PT-symmetry-broken regions are marked by blue dashed lines, with the latter regions bordering the topological phase boundaries.

### Dynamics in the PT-symmetry-unbroken region

We aim to reveal the impacts of the two types of DQPTs on the behaviors of various physical observables and discuss the differences in critical times and critical momenta in non-unitary QWs dynamics. Assumed that the initial state is one of the eigenstates $\left\vert x=0\right\rangle \otimes \left\vert {\psi }_{-}^{i}\right\rangle$ of ${\hat{U}}^{i}$ with the coin parameters $({\theta }_{1}^{i}=\pi /4,{\theta }_{2}^{i}=-\pi /2)$ that are in the PT-symmetry-unbroken region with *ν*^*i*^ = 0. We then implement QWs governed by ${\hat{U}}^{f}$ with various coin parameters $({\theta }_{1}^{f},{\theta }_{2}^{f})$.

**Fig. 2:**
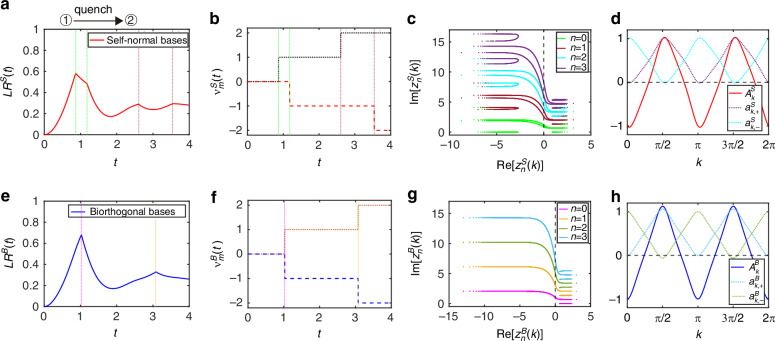
Self-normal and biorthogonal DQPTs during a quench between distinct topological phases in the PT-symmetry-unbroken region. Loschmidt rate of *LR*(*t*) as a function of time in non-unitary QWs with self-normal bases (**a**) and biorthogonal bases (**e**), respectively. DTOPs of *ν*_*m*_(*t*) as a function of time in non-unitary QWs for **b** with self-normal bases and **f** biorthogonal bases, respectively. The green and dark red dot-dashed lines represent the critical times for self-normal DQPTs with ${t}_{c}^{S}\approx 0.869,1.180,2.607$, and 3.54 in (**a**, **b**), corresponding to the Fisher zeros (*n* = 0, 1) crossing the axis in (**c**). The magenta and goldenrod dot-dashed lines represent the critical times for biorthogonal DQPTs, with ${t}_{c}^{B}\approx 1.025$ and 3.075 in (**e**, **f**), corresponding to the Fisher zeros (*n* = 0, 1) crossing the axis in (**g**). **c** Real and imaginary parts of lines of self-normal Fisher zeros ${z}_{n,k}^{S}$ with *n* = 0 (green), *n* = 1 (dark red), *n* = 2 (cyan), *n* = 3 (purple). **g** Real and imaginary parts of lines of biorthogonal Fisher zeros ${z}_{n,k}^{B}$ with *n* = 0 (magenta), *n* = 1 (golden rod), *n* = 2 (dark green), *n* = 3 (deep skyblue). **d** ${A}_{k}^{S}={a}_{k,+}^{S}-{a}_{k,-}^{S}$ as a function of *k*, where self-normal fixed points are at ${k}_{m}^{S}=\{0.189,\pi ,3.331,2\pi \}$ with ${a}_{k+}^{S}=0$ (cyan) and ${k}_{m}^{S}=\{\pi /2,1.761,3\pi /2,4.491\}$ with ${a}_{k,-}^{S}=0$ (purple). **h** ${A}_{k}^{B}={a}_{k,+}^{B}-{a}_{k,-}^{B}$ as a function of *k*, where biorthogonal fixed points are at ${k}_{m}^{S}=\{0,\pi ,2\pi \}$ with ${a}_{k,+}^{B}=0$ (deepskyblue) and ${k}_{m}^{S}=\{1.371,1.766,4.513,4.908\}$ with ${a}_{k,-}^{B}=0$ (darkgreen). In all panels, the loss parameter is *l* = 0.5. The initial state of the QWs is $\left\vert x=0\right\rangle \otimes \left\vert {\psi }_{-}^{i}\right\rangle$ of ${\hat{U}}^{i}$ with the coin parameters $({\theta }_{1}^{i}=\pi /4,{\theta }_{2}^{i}=-\pi /2)$ and the QWs are governed by the final Floquet operator ${\hat{U}}^{f}$ with $({\theta }_{1}^{f}=-\pi /4,{\theta }_{2}^{f}=0)$. All quantities are unitless

First, we consider ${\hat{U}}^{f}$ with the coin parameters $({\theta }_{1}^{f}=-\pi /4,{\theta }_{2}^{f}=0)$. The QW is in the PT-symmetry-unbroken region with *ν*^*f*^ = −2. In Fig. 2a, e, we plot self-normal Loschmidt rate *LR*^*S*^(*t*) and biorthogonal Loschmidt rate *LR*^*B*^(*t*) as a function of time *t*, respectively. It is obvious that the self-normal rate functions exhibit four remarkable peaks, which correspond to four self-normal DTOPs [see Fig. 2b]. The biorthogonal rate functions exhibit two remarkable peaks, which correspond to two biorthogonal DTOPs [see Fig. 2f]. These peaks translate into the logarithmic divergences in the thermodynamic limit. As shown in Fig. 2c, we see that each line of Fisher zeros ${z}_{n,k}^{S}$ for *n* = 0, 1, 2, 3 has two unique crosses on the imaginary axis, yielding two critical times ${t}_{n,c}^{S}$ of self-normal DQPTs. However, in Fig. 2g, each line of Fisher zeros ${z}_{n,k}^{B}$ for *n* = 0, 1, 2, 3 has a unique cross on the imaginary axis, yielding a critical time ${t}_{n,c}^{B}$ of biorthogonal DQPTs. The existence of the two types of DQPTs is indicated by all the aforementioned behaviors.

To reveal these critical points of the two kinds of DQPTs, we analyze the fixed points separately. As shown in Fig. 2d, there are four critical momenta at ${k}_{c}^{S}=\{0.965,2.517,4.107,5.659\}$ satisfying ${a}_{{k}_{c}^{S},+}^{S}={a}_{{k}_{c}^{S},-}^{S}$ between two different kinds of fixed points. Importantly, ${E}_{{k}_{c}^{S}}^{f}$ is degenerate. Hence, there are only two critical time scalings of self-normal DQPTs. In Fig. 2h, there are four critical momenta at ${k}_{c}^{B}=\{0.765,2.377,3.907,5.518\}$ satisfying ${a}_{{k}_{c}^{B},+}^{B}={a}_{{k}_{c}^{B},-}^{B}$. However, ${E}_{{k}_{c}^{B}}^{f}$ is degenerate at all ${k}_{c}^{B}$. Hence, there is only one critical time scaling of biorthogonal DQPTs. We point out that these two types of DQPTs exist distinct critical points in the PT-symmetry-unbroken region. It appears that the biorthogonal method has helped to preserve the system’s symmetry.

**Fig. 3:**
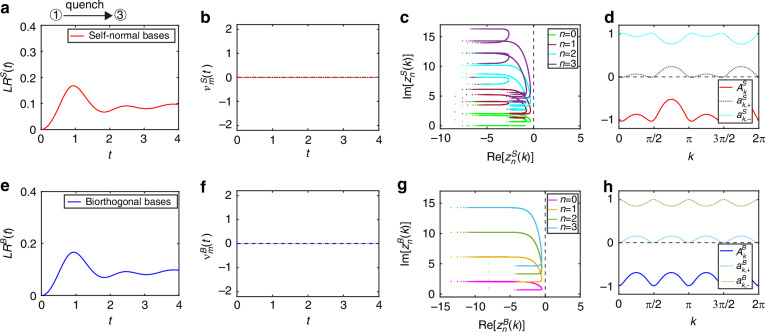
Self-normal and biorthogonal DQPTs during a quench between the same topological phases in the PT-symmetry-unbroken region. Loschmidt rate of *LR*(*t*) as a function of time in non-unitary QWs with self-normal bases (**a**) and biorthogonal bases (**e**), respectively. DTOPs of *ν*_*m*_(*t*) as a function of time in non-unitary QWs for **b** with self-normal bases and **f** biorthogonal bases, respectively. Real and imaginary parts of lines of Fisher zeros *z*_*n*,*k*_ (*n* = 0, 1, 2, 3) with self-normal bases (**c**) and with biorthogonal bases (**g**), respectively. **d** ${A}_{k}^{S}={a}_{k,+}^{S}-{a}_{k,-}^{S}$ as a function of *k*, where self-normal fixed points are at ${k}_{m}^{S}=\{0,0.321,1.251,\pi /2,\pi \}$ with ${a}_{k,+}^{S}=0$ (cyan). **h** ${A}_{k}^{B}={a}_{k,+}^{B}-{a}_{k,-}^{B}$ as a function of *k*, where biorthogonal fixed points are at ${k}_{m}^{B}=\{0,\pi /2,\pi ,2\pi \}$ with ${a}_{k,+}^{B}=0$ (deepskyblue). In all panels, the loss parameter is *l* = 0.5. The initial state of the QWs is $\left\vert x=0\right\rangle \otimes \left\vert {\psi }_{-}^{i}\right\rangle$ of ${\hat{U}}^{i}$ with the coin parameters $({\theta }_{1}^{i}=\pi /4,{\theta }_{2}^{i}=-\pi /2)$ and the QWs are governed by the final Floquet operator ${\hat{U}}^{f}$ with $({\theta }_{1}^{f}=0,{\theta }_{2}^{f}=-\pi /4)$. All quantities are unitless

Second, for comparison, we fix the same initial coin state of ${\hat{U}}^{i}$ with the coin parameters $({\theta }_{1}^{i}=\pi /4,{\theta }_{2}^{i}=-\pi /2)$ and change the final Floquet operator ${\hat{U}}^{f}$ with $({\theta }_{1}^{f}=0,{\theta }_{2}^{f}=-\pi /4)$, where the quench dynamics is between Floquet topological phases with *ν*^*i*^ = 0 and *ν*^*f*^ = 0. As shown in Fig. 3a, e, the self-normal Loschmidt rate and biorthogonal Loschmidt rate are both smooth in time. The self-normal DTOPs ${\nu }_{m}^{S}(t)$ and biorthogonal DTOPs ${\nu }_{m}^{B}(t)$ remain zero in Fig. 3b, f. In addition, each line of Fisher zeros has no crossings on the imaginary time axis as shown in Fig. 3c, g. These behaviors described above indicate the absence of DQPTs, when ${\hat{U}}^{i}$ and ${\hat{U}}^{f}$ have the same winding numbers.

In order to understand the absence of DQPTs, we analyze the fixed points separately. As shown in Fig. 3d, there exist only nine fixed points of the same type at ${a}_{k,+}^{S}=0$, and hence no critical time for self-normal DQPTs. In Fig. 3h, there are five fixed points of the same kind at ${a}_{k,+}^{B}=0$, and hence no critical time for biorthogonal DQPTs. The per- and post-quench systems are in the same phase, no signature of DQPTs is observed. Although the pre- and post-quench systems are in the same phase with no observed signatures of DQPTs, we still identify distinct behaviors of the two types of DQPTs from the perspective of physical quantities.

### Dynamics in the PT-symmetry-broken region

So far, we focus on the distinctions of these two types of DQPTs of critical points in PT-symmetry-unbroken regions. However, it is also necessary to detect the differences between self-normal DQPTs and biorthogonal DQPTs in PT-symmetry-broken regions. To this end, we consider the sudden quench process to ${\hat{U}}^{f}$ belonging to the PT-symmetry-broken regions.

**Fig. 4:**
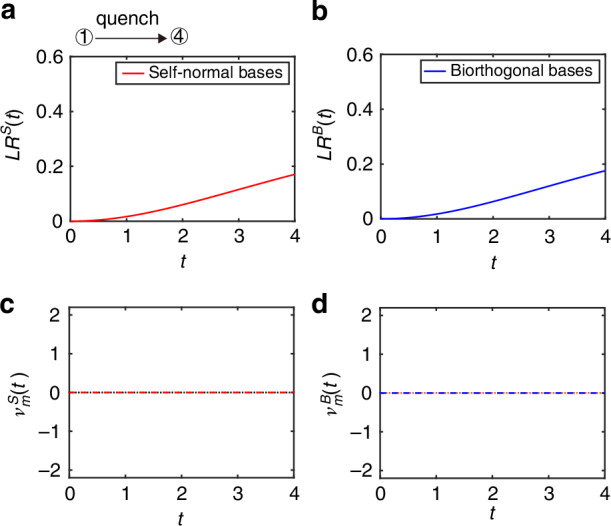
Self-normal and biorthogonal DQPTs during a quench between distinct topological phases in the PT-symmetry-broken region. Loschmidt rate of *LR*(*t*) as a function of time in non-unitary QWs with self-normal bases (**a**) and with biorthogonal bases (**b**), respectively. DTOPs of *ν*(*t*) as a function of time in non-unitary QWs for **c** with self-normal bases and **d** biorthogonal bases, respectively. In all panels, *l* = 0.5, $\xi =\arccos (1/\alpha )$ and $\alpha =(1+\sqrt{1-l})/\left(2\root{4}\of{1-l}\right)$. The initial state of the QWs is $\left\vert x=0\right\rangle \otimes \left\vert {\psi }_{-}^{i}\right\rangle$ of ${\hat{U}}^{i}$ with the coin parameters $({\theta }_{1}^{i}=\pi /4,{\theta }_{2}^{i}=-\pi /2)$ and the QWs are governed by the final Floquet operator ${\hat{U}}^{f}$ with $({\theta }_{1}^{f}=-\pi /2,{\theta }_{2}^{f}=(\pi -\xi )/2)$. All quantities are unitless

**Fig. 5:**
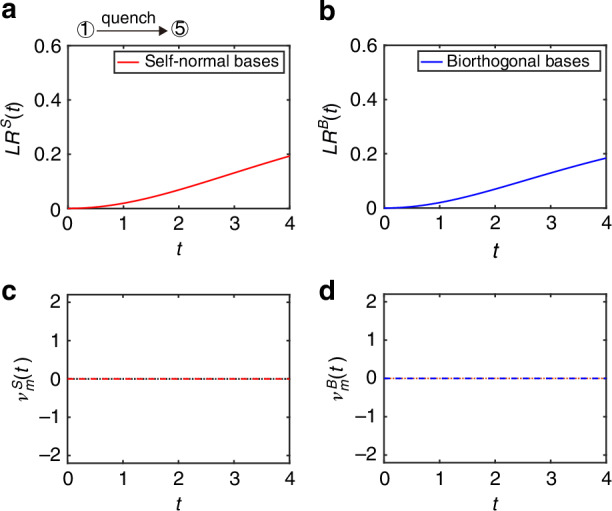
Self-normal and biorthogonal DQPTs during a quench between the same topological phases in the PT-symmetry-broken region. Loschmidt rate of *LR*(*t*) as a function of time in non-unitary QWs with self-normal bases (**a**) and with biorthogonal bases (**b**), respectively. DTOPs of *ν*(*t*) as a function of time in non-unitary QWs for **c** with self-normal bases and **d** biorthogonal bases, respectively. In all panels, *l* = 0.5, $\xi =\arccos (1/\alpha )$ and $\alpha =\left(1+\sqrt{1-l}\right)/\left(2\root{4}\of{1-l}\right)$. The initial state of the QWs is $\left\vert x=0\right\rangle \otimes \left\vert {\psi }_{-}^{i}\right\rangle$ of ${\hat{U}}^{i}$ with the coin parameters $({\theta }_{1}^{i}=\pi /4,{\theta }_{2}^{i}=-\pi /2)$ and the QWs are governed by the final Floquet operator ${\hat{U}}^{f}$ with $({\theta }_{1}^{f}=-(\pi -\xi )/2,{\theta }_{2}^{f}=\pi /2)$. All quantities are unitless

In the third case study, the QWs are governed by the final Floquet operator $({\theta }_{1}^{f}=-\pi /2,{\theta }_{2}^{f}=(\pi -\xi )/2)$ with $\xi =\arccos (1/\alpha )$, which is in different from the topological phase of the initial state. As shown in Fig. 4a, b, the self-normal Loschmidt rate and biorthogonal Loschmidt rate are both smooth in time, which correspond to self-normal DTOPs ${\nu }_{m}^{S}(t)$ and biorthogonal DTOPs ${\nu }_{m}^{B}(t)$ remain zero in Fig. 4c, d, indicating the absence of DQPTs. In the fourth case study, the QWs are governed by the final Floquet operator *U*^*f*^ with (*θ*_1_ = − (*π* − *ξ*)/2, *θ*_2_ = *π*/2), which is in the same topological phase as the initial state. As shown in Fig. 5a, b, the self-normal Loschmidt rate and biorthogonal Loschmidt rate are both smooth in time, corresponding to DTOPs of ${\nu }_{m}^{S}(t)$ and ${\nu }_{m}^{B}(t)$ remain zero in Fig. 5c, d, indicating the absence of DQPTs.

These phenomena demonstrate that both types of DQPTs are absent in the PT-symmetry-broken regions, regardless of the quenching protocol employed. When $\hat{{H}_{f}}$ is in the PT-symmetry-broken region, the corresponding ${E}_{k}^{f}$ becomes imaginary for a certain range of *k*, and the existence of fixed points is no longer guaranteed^35^. More detailed information can be found in Sec. S2 of the Supplementary Material.

### Experimental implementation

**Fig. 6:**
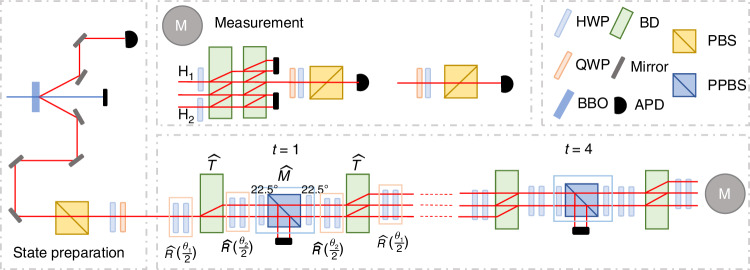
Experimental setup. Photons are generated via spontaneous parametric down conversion in a Type-I non-linear *β*-barium-borate (BBO) crystal. The initial state is prepared by passing horizontally polarized photons, filtered by a polarizing beam splitter (PBS), through a half-wave plate (HWP) and a quarter-wave plate (QWP) with certain setting angles. The conditional shift operation $\hat{T}$ is implemented using a beam displacer (BD), while the coin operator $\hat{R}$ is realized by two HWPs. The non-unitary loss operator $\hat{M}$ is implemented by introducing photon loss through a sandwich-type configuration consisting of two HWPs and a partially polarizing beam splitter (PPBS). Two types of measurements, projective and interference-based measurements, are performed to construct the evolved state before the photons are detected by avalanche photodiodes (APDs)

We perform an experiment to validate our theoretical prediction regarding the connection between self-normal and biorthogonal DQPTs. As illustrated in Fig. 6, we encode the coin and position states into the polarization and spatial modes of single photons, respectively. The conditional shift operation $\hat{T}$ is implemented using a beam displacer (BD), which transmits vertically polarized photons directly and shifts the horizontally polarized photons by a lateral displacement. The coin operator $\hat{R}$ is realized by two half-wave plates (HWPs). At each step, the non-unitary loss operator $\hat{M}$ is implemented by a sandwich-type setup involving two HWPs at 22.5^∘^ and a partially polarizing beam splitter (PPBS). This setup allows photons in the polarization state $| -\left.\right\rangle$ to be reflected with probability *l*, while the remaining photons continue to evolve under the quantum-walk dynamics.

By changing the setting angles of the wave plates to different (*θ*_1_, *θ*_2_), the non-unitary QW can be either in the PT-symmetry-unbroken or PT-symmetry-broken regions. To achieve the quench process, we initialize the state of the dynamics in the eigenstate of the initial Hamiltonian with the parameters of (${\theta }_{1}^{i},{\theta }_{2}^{i}$). Without any time delay, the system evolves under the final Hamiltonian with the parameters of (${\theta }_{1}^{f},{\theta }_{2}^{f}$).

Regarding the self-normal DQPTs, by changing the setting angles of the wave plates, we prepare the initial state on a pure state $| {\psi }_{k,-}^{R,i}\left.\right\rangle$. Afterwards, we realize the evolution process of ${\hat{U}}^{f}$ using the combination of different optical components. By experimentally measuring $\left\langle \right.{\psi }_{{x}_{2}}(t)| {\hat{\sigma }}_{j}| {\psi }_{{x}_{2}}(t)\left.\right\rangle$^31^, we reconstruct the density matrix of the evolved state as

16 $$\begin{array}{lll}\rho (k,t)&=&| {\varPsi }_{k}^{R}(t)\left.\right\rangle \left\langle \right.{\varPsi }_{k}^{R}(t)| \\ &=&\frac{1}{2}\mathop{\sum }\limits_{j=0}^{3}\mathop{\sum}\limits_{{x}_{1},{x}_{2}}{e}^{-ik({x}_{1}-{x}_{2})}\left\langle \right.{\psi }_{{x}_{2}}(t)| {\hat{\sigma }}_{j}| {\psi }_{{x}_{1}}(t)\left.\right\rangle {\hat{\sigma }}_{j}\end{array}$$

Here, ${\hat{\sigma }}_{j}$ (*j* = 0, *x*, *y*, *z*) denote the Pauli matrix, and $| {\psi }_{x}\left.\right\rangle$ is the coin state on site *x* at the *t*th step. We then obtain $L{E}_{k}^{S}(t)=\,{\text{Tr}}\,[\rho (k,0)\rho (k,t)]/\,{\text{Tr}}\,[\rho (k,t)]$. By applying this result to Eq. (5), we derive the self-normal Loschmidt rate. The vectors of the evolved state $|\varPsi^R_k(t)\rangle$ can also be extracted from the reconstructed density matrix *ρ*(*k*, *t*). This allows us to determine the self-normal DTOPs (see Sec. S3 of the Supplementary Material).

In the context of biorthogonal DQPTs, we reconstruct both the right and left eigenstates, $| {\tilde{\psi }}_{k,-}^{R,i}\left.\right\rangle$ and $| {\tilde{\psi }}_{k,-}^{L,i}\left.\right\rangle$, of the initial Hamiltonian. Using the experimentally measured *ρ*(*k*, *t*), we obtain the non-Hermitian density matrix $\tilde{\rho }(k,t)=| {\tilde{\varPsi }}_{k}^{R}(t)\left.\right\rangle \left\langle \right.{\tilde{\varPsi }}_{k}^{L}(t)|$ as

17 $$\tilde{\rho }(k,t)=\frac{\rho (k,t){\sum }_{\omega = \pm }\left| {\tilde{\psi }}_{k,\omega }^{L,f}\left.\right\rangle \left\langle \right.{\tilde{\psi }}_{k,\omega }^{L,f}\right| }{\,{\text{Tr}}\,\left[\rho (k,t){\sum }_{\omega = \pm }\left| {\tilde{\psi }}_{k,\omega }^{L,f}\left.\right\rangle \left\langle \right.{\tilde{\psi }}_{k,\omega }^{L,f}\right| \right]}$$

We then obtain $L{E}_{k}^{B}(t)=\,{\rm{Tr}}\,[\tilde{\rho }(k,0)\tilde{\rho }(k,t)]/\,{\rm{Tr}}\,[\tilde{\rho }(k,t)]$. By applying this result to Eq. (10), we obtain the biorthogonal Loschmidt rate. Using the reconstructed $| {\tilde{\psi }}_{k,-}^{L,i}\left.\right\rangle$ and $| {\tilde{\varPsi }}_{k}^{R}(t)\left.\right\rangle$, we further determine the biorthogonal DTOPs.

**Fig. 7:**
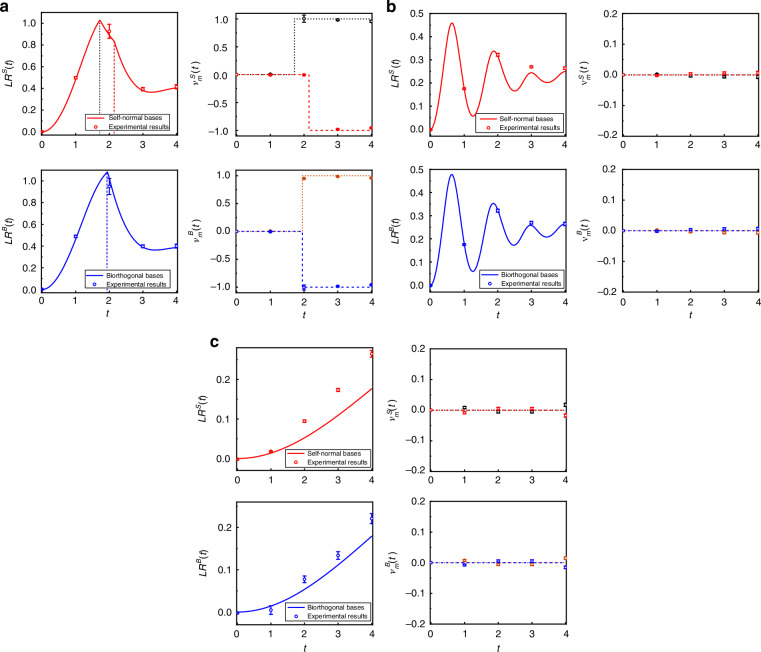
Experimental results. Measured Loschmidt rate *LR*(*t*) and DTOPs of *ν*_*m*_(*t*) as functions of time for QWs governed by $\hat{U}^f$ with **a** $({\theta }_{1}^{f}=-\pi /3,{\theta }_{2}^{f}=\pi /5)$ (*ν*^*f*^ = −2) and **b** $({\theta }_{1}^{f}=-\pi /4,{\theta }_{2}^{f}=-5\pi /16)$ (*ν*^*f*^ = 0) in the PT-symmetry-unbroken region, respectively. **c** Loschmidt rate and DTOPs of the QW governed by $\hat{U}^f$ with $({\theta }_{1}^{f}=-\pi /2,{\theta }_{2}^{f}=\pi -\xi /2)$ in the PT-symmetry-broken region. The initial state of the QWs is $| 0\left.\right\rangle \otimes | {\psi }_{-}^{i}\left.\right\rangle$, corresponding to the ground eigenstate of $\hat{U}^i$ with coin parameters $({\theta }_{1}^{i}=\pi /4,{\theta }_{2}^{i}=-\pi /2)$ and *ν*^*i*^ = 0. The loss parameter is fixed at *l* = 0.36. Theoretical predictions are shown by lines, and corresponding experimental results are shown by symbols. Error bars indicate the statistical uncertainty, which is obtained based on assuming Poissonian statistics

As shown in Fig. 7, we present the experimental results for both self-normal and biorthogonal DQPTs under different quench dynamics. Fixing the loss parameter at *l* = 0.36, we first consider a quench from *ν*^*i*^ = 0 with $({\theta }_{1}^{i}=\pi /4,{\theta }_{2}^{i}=-\pi /2)$ to *ν*^*f*^ = −2 with $({\theta }_{1}^{f}=-\pi /3,{\theta }_{2}^{f}=\pi /5)$ in the PT-symmetry-unbroken region. The corresponding results are shown in Fig. 7a. The self-normal Loschmidt rate and DTOPs exhibit two distinct critical times associated with DQPTs, whereas the biorthogonal condition shows only a single critical time. For comparison, we characterize a quench between the same Floquet topological phases with *ν*^*i*^ = *ν*^*f*^ = 0. Keeping the initial parameters unchanged, the evolution is governed by *U*^*f*^ with $({\theta }_{1}^{f}=-\pi /4,{\theta }_{2}^{f}=-5\pi /16)$. As shown in Fig. 7b, the Loschmidt rate remains smooth and the DTOPs stay at zero, indicating the absence of DQPTs in both self-normal and biorthogonal cases. We further investigate a quench from *ν*_*i*_ = 0 with $({\theta }_{1}^{i}=\pi /4,{\theta }_{2}^{i}=-\pi /2)$ to *ν*^*f*^ = −2 with $({\theta }_{1}^{f}=-\pi /2,{\theta }_{2}^{f}=(\pi -\xi )/2)$ in the PT-symmetry-broken region. As shown in Fig. 7c, both the self-normal and biorthogonal Loschmidt rates are smooth in time and the corresponding DTOPs remain zero, which all signals the absence of the DQPTs. All the experimental results agree with their theoretical results, which infers our theoretical predictions.

## Discussion

In conclusion, based on the theoretical frameworks of self-normal bases and biorthogonal bases, we have discussed the concepts of self-normal and biorthogonal DQPTs on the non-unitary quench dynamics. To understand these two kinds of DQPTs, we theoretically investigate the rate functions, DTOPs, and Fisher zeros under the distinct theoretical frameworks, respectively. Our exploration focuses on the properties of these two kinds of DQPTs in the one-dimensional non-Hermitian QWs, which exist distinct topological phases in PT-symmetry-unbroken regions.

Although the physical origins of these two kinds of DQPTs are different, both of them can be found when the system is quenched from distinct non-Hermitian topological phases in the PT-symmetry-unbroken region. We have shown that self-normal and biorthogonal DQPTs are signified by the logarithmic divergence of the Loschmidt rate functions at their critical times, corresponding to integer jumps in the DTOPs. However, it appears that the biorthogonal method has helped to preserve the system’s symmetry in PT-symmetry-unbroken regions. This is also an area that we need to explore further.

Furthermore, we have discussed how these two types of DQPTs are distinguished by critical times on each line of Fisher zeros and critical momenta with different fixed points. We point out that the number and location of critical momenta and critical times are different for these two kinds of DQPTs. Finally, we present an experiment to observe self-normal DQPTs and biorthogonal DQPTs via discrete-time non-Hermitian QWs.

The self-normal and biorthogonal frameworks offer distinct yet complementary insights into the nonequilibrium dynamics of non-Hermitian systems. In particular, our simultaneous observation of both types of DQPTs highlights the versatility of QWs and our photonics platform for exploring rich dynamical behavior that goes beyond the Hermitian paradigm. Furthermore, when starting from a known topological phase, the emergence of these two types of DQPTs under quench dynamics serves as a powerful tool for identifying and characterizing topological phase boundaries in non-Hermitian systems^46^. Their sensitivity to initial conditions and system parameters across different phases also opens promising avenues for enhanced control in quantum simulation and precision quantum sensing^47–49^. Looking forward, the joint observation of self-normal and biorthogonal DQPTs paves the way for systematic studying their interrelations and evaluating their robustness against disorder, decoherence, and interactions. It also inspires further extensions to higher-dimensional non-Hermitian models, thereby expanding the landscape of dynamical quantum phase transitions in complex quantum systems.

## Materials and methods

### Self-normal Fisher zeros and fixed points

Similar to the disappearance of the partition function at equilibrium phase transitions, the lines of self-normal Fisher zeros here are determined by the vanishing of the self-normal Loschmidt echo, i.e., $L{E}_{k}^{S}(t)=0$. This condition signifies a complete destructive interference between the initial and time-evolved states. The self-normal Fisher zeros^2,50,51^ can be derived as follows:

18 $$\begin{array}{lll}&&\langle {\psi }_{k,-}^{R,i}| {\varPsi }_{k}^{R}(t)\rangle =0\\ &&\ \Rightarrow \ \cos \left({E}_{k}^{f}t\right)-i\sin ({E}_{k}t){A}_{k}^{S}=0\\ &&\ \Rightarrow \ {z}_{k}^{S}=\frac{1}{2{E}_{k}^{f}}\ln (-1)+\frac{1}{2{E}_{k}^{f}}\ln \left(\frac{1+{A}_{k}^{S}}{1-{A}_{k}^{S}}\right)\end{array}$$

Thus, we can arrive at the expression for the self-normal Fisher zeros ${z}_{n,k}^{S}$, which is detailed as follows:

19 $${z}_{n,k}^{S}=i\left(n+\frac{1}{2}\right)\frac{\pi }{{E}_{k}^{f}}+\frac{1}{{E}_{k}^{f}}{\tanh }^{-1}\left({A}_{k}^{S}\right)$$

where *n* is an integer. Meanwhile, ${A}_{k}^{S}={a}_{k+}^{S}-{a}_{k-}^{S}$ with ${a}_{k,+}^{S}=\langle {\psi }_{k,-}^{R,i}| {\tilde{\psi }}_{k,+}^{R,f}\rangle \langle {\tilde{\psi }}_{k,+}^{L,f}| {\psi }_{k,-}^{R,i}\rangle$ and ${a}_{k,-}^{S}=\langle {\psi }_{k-}^{R,i}| {\tilde{\psi }}_{k,-}^{R,f}\rangle \langle {\tilde{\psi }}_{k,-}^{L,f}| {\psi }_{k,-}^{R,i}\rangle$ when the system is quenched between different topological phases in the PT-symmetry-unbroken region, self-normal fixed points^52^ with ${a}_{k,+}^{S}=0$ and those with ${a}_{k,-}^{S}=0$ always emerge in pairs. Since ${A}_{k}^{S}$ are continuous functions of ${k}_{m}^{S}$, there must be at least one critical momentum satisfying ${a}_{k,+}^{S}={a}_{k,-}^{S}$ between two fixed points of different kinds.

### Biorthogonal Fisher zeros and fixed points

Similarly, the lines of biorthogonal Fisher zeros are given by the biorthogonal Loschmidt echo, i.e., $L{E}_{k}^{B}(t)=0$. From Eq. (9), one can obtain the biorthogonal Fisher zeros as follows:

20 $$\begin{array}{lll}&&\langle {\tilde{\psi }}_{k,-}^{L,i}| {\tilde{\varPsi }}_{k}^{R}(t)\rangle =0\\ &&\ \Rightarrow \ \cos \left({E}_{k}^{f}t\right)-i\sin \left({E}_{k}^{f}t\right){A}_{k}^{B}=0\\ &&\ \Rightarrow \ {z}_{k}^{B}=\frac{1}{2{E}_{k}^{f}}\ln (-1)+\frac{1}{2{E}_{k}^{f}}\ln \left(\frac{1+{A}_{k}^{B}}{1-{A}_{k}^{B}}\right)\end{array}$$

Hence, the line of biorthogonal Fisher zeros ${z}_{n,k}^{B}$ is given by

21 $${z}_{n,k}^{B}=i\left(n+\frac{1}{2}\right)\frac{\pi }{{E}_{k}^{f}}+\frac{1}{{E}_{k}^{f}}{\tanh }^{-1}\left({A}_{k}^{B}\right)$$

Here *n* is an integer. ${A}_{k}^{B}={a}_{k,+}^{B}-{a}_{k,-}^{B}$ with ${a}_{k,+}^{B}=\langle {\tilde{\psi }}_{k,-}^{L,i}| {\tilde{\psi }}_{k,+}^{R,f}\rangle \langle {\tilde{\psi }}_{k,+}^{L,f}| {\tilde{\psi }}_{k,-}^{R,i}\rangle$ and ${a}_{k,-}^{B}=\langle {\tilde{\psi }}_{k,-}^{L,i}| {\tilde{\psi }}_{k,-}^{R,f}\rangle \langle {\tilde{\psi }}_{k,-}^{L,f}| {\tilde{\psi }}_{k,-}^{R,i}\rangle$. When the system is quenched between different topological phases in the PT-symmetry-unbroken region, biorthogonal fixed points with ${a}_{k,+}^{B}=0$ and those with ${a}_{k,-}^{B}=0$ always emerge in pairs. Since ${A}_{k}^{B}$ are continuous functions of ${k}_{m}^{B}$, there must be at least one critical momentum satisfying ${a}_{k,+}^{B}={a}_{k,-}^{B}$ between two fixed points of different kinds.

## Supplementary information

Supplementary Information

## Data Availability

Any related background information, data and code not mentioned in the text and other findings of this study are available from the corresponding author upon reasonable request.
