# Supplementary material for: Self-normal and biorthogonal dynamical quantum phase transitions in non-Hermitian quantum walks
Source: Light Sci Appl. 2025 Jul 26;14:253. doi: 10.1038/s41377-025-01919-6 (PMC12297308; doi:10.1038/s41377-025-01919-6)
Supplement: Supplementary file 1 — Supplementary Information [file 41377_2025_1919_MOESM1_ESM.pdf]

Supplementary Information for Self-Normal and Biorthogonal Dynamical Quantum Phase Transitions in Non-Hermitian Quantum Walks

Haiting Zhang[1,†], Kunkun Wang[2,†], Lei Xiao[3], Peng Xue[1,*]
[1]Beijing Computational Science Research Center, Beijing 100193, China
[2]School of Physics and Optoelectronic Engineering, Anhui University, Hefei 230601, China
[3]Key Laboratory of Quantum Materials and Devices of Ministry of Education, School of Physics, Southeast University, Nanjing 211189, China
[†]These authors contributed equally: Haiting Zhang, Kunkun Wang
[*]Correspondence: Peng Xue (gnep.eux@gmail.com)

In this supplementary material, we will show more details to support the discussion in the main text. In Sec. S1, we show the detailed theoretical calculations of self-normal and biorthogonal physical quantities. In Sec. S2, we present more detailed information on the two types of DQPTs between different topological phases in the PT-symmetry-broken region. In Sec. S3, we provide more detailed information on experimental proposal.

S1. DETAILED THEORETICAL CALCULATIONS

A. Detailed calculations of the self-normal related physical quantities

The self-normal Loschmidt echo is then written by $LE^S(t)=\prod_k LE_k^S(t)$ in detail with

$$\begin{aligned} LE_k^S(t) &= \frac{\langle\Psi_k^R(t)|\Psi_k^R(0)\rangle\langle\Psi_k^R(0)|\Psi_k^R(t)\rangle}{\langle\Psi_k^R(t)|\Psi_k^R(t)\rangle\langle\Psi_k^R(0)|\Psi_k^R(0)\rangle} \\ &= \frac{\langle\Psi_k^R(t)|\psi_{k,-}^{R,i}\rangle\langle\psi_{k,-}^{R,i}|\Psi_k^R(t)\rangle}{\langle\Psi_k^R(t)|\Psi_k^R(t)\rangle\langle\psi_{k,-}^{R,i}|\psi_{k,-}^{R,i}\rangle} \\ &= \frac{|\langle\psi_{k,-}^{R,i}|\Psi_k^R(t)\rangle|^2}{\langle\Psi_k^R(t)|\Psi_k^R(t)\rangle}. \end{aligned} \tag{S1}$$

Then, we can calculate the numerator

$$\begin{aligned} \langle\psi_{k,-}^{R,i}|\Psi_k^R(t)\rangle &= \langle\psi_{k,-}^{R,i}|e^{-i\hat{H}_k^f t}|\psi_{k,-}^{R,i}\rangle \\ &= e^{-iE_k^f t}\langle\psi_{k,-}^{R,i}|\tilde{\psi}_{k,+}^{R,f}\rangle\langle\tilde{\psi}_{k,+}^{L,f}|\psi_{k,-}^{R,i}\rangle + e^{iE_k^f t}\langle\psi_{k,-}^{R,i}|\tilde{\psi}_{k,-}^{R,f}\rangle\langle\tilde{\psi}_{k,-}^{L,f}|\psi_{k,-}^{R,i}\rangle, \end{aligned} \tag{S2}$$

and the denominator

$$\begin{aligned} \langle\Psi_k^R(t)|\Psi_k^R(t)\rangle &= |e^{-i\hat{H}_k^f t}|\Psi_k^R(0)\rangle|^2 \\ &= |e^{-iE_k^f t}|\tilde{\psi}_{k,+}^{R,f}\rangle\langle\tilde{\psi}_{k,+}^{L,f}|\psi_{k,-}^{R,i}\rangle + e^{iE_k^f t}|\tilde{\psi}_{k,-}^{R,f}\rangle\langle\tilde{\psi}_{k,-}^{L,f}|\psi_{k,-}^{R,i}\rangle|^2. \end{aligned} \tag{S3}$$

To obtain the form of self-normal dynamical phase given in the main text, we introduce the self-normal time-dependent normalized state as

$$|\Phi_k^R(t)\rangle = \frac{|\Psi_k^R(t)\rangle}{\sqrt{\langle\Psi_k^R(t)|\Psi_k^R(t)\rangle}}. \tag{S4}$$

Since $\langle\Phi_k^R(t)|\Phi_k^R(t)\rangle=1$, we have $\frac{d}{dt}\langle\Phi_k^R(t)|\Phi_k^R(t)\rangle=0$, implying that $\langle\Phi_k^R(t)|\frac{d}{dt}|\Phi_k^R(t)\rangle$ is purely imaginary. A real self-normal dynamical phase is then defined in terms of the normalized state as

$$\begin{aligned} \phi_k^{S,dyn}(t) &= -i\int_0^t dt'\langle\Phi_k^R(t')|\frac{d}{dt'}|\Phi_k^R(t')\rangle \\ &= -i\int_0^t dt'\frac{\langle\Psi_k^R(t')|}{\sqrt{\langle\Psi_k^R(t')|\Psi_k^R(t')\rangle}}\frac{d}{dt'}\frac{|\Psi_k^R(t')\rangle}{\sqrt{\langle\Psi_k^R(t')|\Psi_k^R(t')\rangle}} \\ &= -i\int_0^t dt'\frac{\langle\Psi_k^R(t')|}{\sqrt{\langle\Psi_k^R(t')|\Psi_k^R(t')\rangle}}\frac{\frac{d}{dt'}|\Psi_k^R(t')\rangle}{\sqrt{\langle\Psi_k^R(t')|\Psi_k^R(t')\rangle}} + \frac{\langle\Psi_k^R(t')|\Psi_k^R(t')\rangle}{\sqrt{\langle\Psi_k^R(t')|\Psi_k^R(t')\rangle}}\frac{d}{dt'}(\frac{1}{\sqrt{\langle\Psi_k^R(t')|\Psi_k^R(t')\rangle}}) \\ &= -\int_0^t dt'\frac{\langle\Psi_k^R(t')|i\frac{d}{dt'}|\Psi_k^R(t')\rangle}{\langle\Psi_k^R(t')|\Psi_k^R(t')\rangle} + \frac{i}{2}\ln[\frac{\langle\Psi_k^R(t')|\Psi_k^R(t')\rangle}{\langle\Psi_k^R(0)|\Psi_k^R(0)\rangle}]. \end{aligned} \tag{S5}$$

Using $\langle\Psi_k^R(0)|\Psi_k^R(0)\rangle=1$ and the Schrödinger equation $i\frac{d}{dt}|\Psi_k^R(t)\rangle=\hat{H}_k^f|\Psi_k^R(t)\rangle$, the self-normal dynamical phase is found to be

$$\phi_k^{S,dyn}(t) = -\int_0^t dt'\frac{\langle\Psi_k^R(t')|\hat{H}_k^f|\Psi_k^R(t')\rangle}{\langle\Psi_k^R(t')|\Psi_k^R(t')\rangle} + \frac{i}{2}\ln[\langle\Psi_k^R(t')|\Psi_k^R(t')\rangle]. \tag{S6}$$

B. Detailed calculations of the biorthogonal related physical quantities

The biorthogonal Loschmidt echo is then given by $LE^B(t) = \prod_k LE_k^B(t)$, with the detailed expression as follows:

$$
\begin{aligned}
LE_k^B(t) &= \frac{\langle\tilde{\Psi}_k^L(t)|\tilde{\Psi}_k^R(0)\rangle\langle\tilde{\Psi}_k^L(0)|\tilde{\Psi}_k^R(t)\rangle}{\langle\tilde{\Psi}_k^L(t)|\tilde{\Psi}_k^R(t)\rangle\langle\tilde{\Psi}_k^L(0)|\tilde{\Psi}_k^R(0)\rangle} \\
&= \frac{\langle\tilde{\Psi}_k^L(t)|\tilde{\psi}_{k,-}^{R,i}\rangle\langle\tilde{\psi}_{k,-}^{L,i}|\tilde{\Psi}_k^R(t)\rangle}{\langle\tilde{\Psi}_k^L(t)|\tilde{\Psi}_k^R(t)\langle\tilde{\psi}_{k,-}^{L,i}|\tilde{\psi}_{k,-}^{R,i}\rangle} \\
&= \frac{|\langle\tilde{\psi}_{k,-}^{L,i}|\tilde{\Psi}_k^R(t)\rangle|^2}{\langle\tilde{\Psi}_k^L(t)|\tilde{\Psi}_k^R(t)},
\end{aligned}
\tag{S7}
$$

where $\langle\tilde{\psi}_{k,-}^{L,i}|\tilde{\psi}_{k,-}^{R,i}\rangle = 1$. Then, we can calculate the numerators

$$
\begin{aligned}
\langle\tilde{\psi}_{k,-}^{L,i}|\tilde{\Psi}_k^R(t)\rangle &= \langle\tilde{\psi}_{k,-}^{L,i}|(c_{k,+}(t)|\tilde{\psi}_{k,-}^{R,i}\rangle| + c_{k,-}(t)|\tilde{\psi}_{k,-}^{R,i}\rangle) \\
&= c_{k,-}(t) \\
&= \langle\tilde{\psi}_{k,-}^{L,i}|e^{-i\hat{H}_k^f t}|\tilde{\psi}_{k,-}^{R,i}\rangle \\
&= e^{-iE_k^f t}\langle\tilde{\psi}_{k,-}^{L,i}|\tilde{\psi}_{k,+}^{R,f}\rangle\langle\tilde{\psi}_{k,+}^{L,f}|\tilde{\psi}_{k,-}^{R,i}\rangle + e^{iE_k^f t}\langle\tilde{\psi}_{k,-}^{L,i}|\tilde{\psi}_{k,-}^{R,f}\rangle\langle\tilde{\psi}_{k,-}^{L,f}|\tilde{\psi}_{k,-}^{R,i}\rangle,
\end{aligned}
\tag{S8}
$$

$$
\begin{aligned}
\langle\tilde{\Psi}_k^L(t)|\tilde{\psi}_{k,-}^{R,i}\rangle &= (c_{k,+}^*(t)\langle\tilde{\psi}_{k,+}^{L,i}| + c_{k,-}^*(t)\langle\tilde{\psi}_{k,-}^{L,i}|)|\tilde{\psi}_{k,-}^{R,i}\rangle \\
&= c_{k,-}^*(t),
\end{aligned}
\tag{S9}
$$

and the denominator

$$
\begin{aligned}
\langle\tilde{\Psi}_k^L(t)|\tilde{\Psi}_k^R(t)\rangle &= (\langle\tilde{\psi}_{k,-}^{L,i}|c_{k,-}^*(t) + \langle\tilde{\psi}_{k,-}^{L,i}|c_{k,-}^*(t)) \times (c_{k,-}(t)|\tilde{\psi}_{k,-}^{R,i}\rangle + c_{k,-}(t)|\tilde{\psi}_{k,-}^{R,i}\rangle) \\
&= |c_{k,-}(t)|^2 + |c_{k,-}(t)|^2,
\end{aligned}
\tag{S10}
$$

where $c_{k,+}(t) = \langle\tilde{\psi}_{k,+}^{L,i}|\tilde{\Psi}_k^R(t)\rangle$ and $c_{k,-}(t) = \langle\tilde{\psi}_{k,-}^{L,i}|\tilde{\Psi}_k^R(t)\rangle$.

To obtain the form of biorthogonal dynamical phase given in the main text, we introduce the biorthogonal time-dependent normalized states as

$$
|\tilde{\Phi}_k^R(t)\rangle = \frac{|\tilde{\Psi}_k^R(t)\rangle}{\sqrt{\langle\tilde{\Psi}_k^L(t)|\tilde{\Psi}_k^R(t)\rangle}},
\tag{S11}
$$

and

$$
\langle\tilde{\Phi}_k^L(t)| = \frac{\langle\tilde{\Psi}_k^L(t)|}{\sqrt{\langle\tilde{\Psi}_k^L(t)|\tilde{\Psi}_k^R(t)\rangle}}.
\tag{S12}
$$

Since $\langle\tilde{\Phi}_k^L(t)|\tilde{\Phi}_k^R(t)\rangle = 1$, we have $\frac{d}{dt}\langle\tilde{\Phi}_k^L(t)|\tilde{\Phi}_k^R(t)\rangle = 0$, implying that $\langle\tilde{\Phi}_k^L(t)|\frac{d}{dt}|\tilde{\Phi}_k^R(t)\rangle$ is purely imaginary. A real biorthogonal dynamical phase is then defined in terms of the normalized state as

$$
\begin{aligned}
\phi_k^{B,dyn}(t) &= -i\int_0^t dt'\langle\tilde{\Phi}_k^L(t')|\frac{d}{ds}|\tilde{\Phi}_k^R(t')\rangle \\
&= -i\int_0^t dt'\frac{\langle\tilde{\Psi}_k^L(t')|}{\sqrt{\langle\tilde{\Psi}_k^L(t')|\tilde{\Psi}_k^R(t')\rangle}}\frac{d}{dt'}\frac{|\tilde{\Psi}_k^R(t')\rangle}{\sqrt{\langle\tilde{\Psi}_k^L(t')|\tilde{\Psi}_k^R(t')\rangle}} \\
&= -i\int_0^t dt'\frac{\langle\tilde{\Psi}_k^L(t')|}{\sqrt{\langle\tilde{\Psi}_k^L(t')|\tilde{\Psi}_k^R(t')\rangle}}\frac{\frac{d}{dt'}|\tilde{\Psi}_k^L(t')\rangle}{\sqrt{\langle\tilde{\Psi}_k^L(t')|\tilde{\Psi}_k^R(t')\rangle}} + \frac{\langle\tilde{\Psi}_k^L(t')|\tilde{\Psi}_k^R(t')\rangle}{\sqrt{\langle\tilde{\Psi}_k^L(t')|\tilde{\Psi}_k^R(t')\rangle}}\frac{d}{dt'}(\frac{1}{\sqrt{\langle\tilde{\Psi}_k^L(t')|\tilde{\Psi}_k^R(t')\rangle}}) \\
&= -\int_0^t dt'\frac{\langle\tilde{\Psi}_k^L(t')|i\frac{d}{dt'}|\tilde{\Psi}_k^R(t')\rangle}{\langle\tilde{\Psi}_k^L(t')|\tilde{\Psi}_k^R(t')\rangle} + \frac{i}{2}\ln[\frac{\langle\tilde{\Psi}_k^L(t')|\tilde{\Psi}_k^R(t')\rangle}{\langle\tilde{\Psi}_k^L(0)|\tilde{\Psi}_k^R(0)\rangle}].
\end{aligned}
\tag{S13}
$$

Using $\langle\tilde{\Psi}_k^L(0)|\tilde{\Psi}_k^R(0)\rangle = 1$ and the Schrödinger equation $i\frac{d}{dt}|\tilde{\Psi}_k^R(t)\rangle = \hat{H}_k^f|\tilde{\Psi}_k^R(t)\rangle$, the biorthogonal dynamical phase is found to be

$$\phi_k^{B,dyn}(t) = -\int_0^t dt' \frac{\langle\tilde{\Psi}_k^L(t')|\hat{H}_k^f|\tilde{\Psi}_k^R(t')\rangle}{\langle\tilde{\Psi}_k^L(t')|\tilde{\Psi}_k^R(t')\rangle} + \frac{i}{2}\ln[\langle\tilde{\Psi}_k^L(t)|\tilde{\Psi}_k^R(t)\rangle]. \tag{S14}$$

C. Outline of key physical quantities

In the theoretical part, various key physical quantities are introduced to characterize the two types of DQPTs. For clarity, we summarize key physical quantities in Table S1.

TABLE S1. Outline of the concepts introduced in Result: key quantities characterizing self-normal DQPTs and biorthogonal DQPTs.

Concepts	Self-normal Expressions	Biorthogonal Expressions	Annotations
Initial state	$\lvert\Psi_k^R(0)\rangle = \lvert\psi_{k,-}^{R,i}\rangle$	$\lvert\tilde{\Psi}_k^R(0)\rangle = \lvert\tilde{\psi}_{k,-}^{R,i}\rangle$	"-" ground state
Return amplitude	$LA_k^S(t) = \langle\psi_{k,-}^{R,i}\lvert\Psi_k^R(t)\rangle$	$LA_k^B(t) = \langle\tilde{\psi}_{k,-}^{L,i}\lvert\tilde{\Psi}_k^R(t)\rangle$	Dynamical partition function
Loschmidt echo	$LE_k^S(t) = \frac{\lvert\langle\psi_{k,-}^{R,i}\lvert\Psi_k^R(t)\rangle\rvert^2}{\langle\Psi_k^R(t)\lvert\Psi_k^R(t)\rangle}$	$LE_k^B(t) = \frac{\lvert\langle\tilde{\psi}_{k,-}^{L,i}\lvert\tilde{\Psi}_k^R(t)\rangle\rvert^2}{\langle\tilde{\Psi}_k^L(t)\lvert\tilde{\Psi}_k^R(t)}$	Return probability
Rate function	$LR^S(t) = -\frac{1}{2\pi}\int_0^{2\pi} dk\ln[LE_k^S(t)]$	$LR^B(t) = -\frac{1}{2\pi}\int_0^{2\pi} dk\ln[LE_k^B(t)]$	Dynamical free energy
Crititical times	$t_{n,c}^S = (2n-1)t_0^S/2$	$t_{n,c}^B = (2n-1)t_0^B/2$	DQPTs happen at each t_c
Crititical momenta	k_c^S	k_c^B	$k_c \in [k_m, k_{m+1}]$
Fisher zeros	$\mathrm{Re}[z_{n,k_c^S}] = 0$	$\mathrm{Re}[z_{n,k_c^B}] = 0$	Complex plane ($it \to z = \tau + it \in \mathbb{C}$)
Fixed points	$a_{k,+}^S = 0$ or $a_{k,-}^S = 0$	$a_{k,+}^B = 0$ or $a_{k,-}^B = 0$	k_m
DTOPs	$\nu_m^S(t) = \frac{1}{2\pi}\int_{k_m^S}^{k_{m+1}^S} dk[\partial_k\phi_k^{S,G}(t)]$	$\nu_m^B(t) = \frac{1}{2\pi}\int_{k_m^B}^{k_{m+1}^B} dk[\partial_k\phi_k^{B,G}(t)]$	Dynamical winding number

S2. MORE DETAILED INFORMATION ON THE TWO TYPES OF DQPTS

The self-normal DQPTs and biorthogonal DQPTs are unable to happen between different topological phases in the PT-symmetry-broken region. The occurrence of DQPT is related to the existence of critical momentum between two different kinds of fixed points in momentum space. DQPTs can happen when there exists a critical momentum such that the initial state evolves to an orthogonal state at certain critical times. This condition is often associated with the appearance of Fisher zeros in the complex plane. However, when E_k^f is imaginary in the PT-symmetry-broken region, the instantaneous state vector asymptotically approaches the North Pole on the Bloch sphere. The system always relaxes to the steady state at long times, and there is no dynamic fixed point [1–3]. Thus, we are unable to observe these two types of DQPTs in the PT-symmetry-broken region.

Although the two types of DQPTs do not exist, there are still differences in the specific numerical values. To further clarify this point, we fix the initial state and take another quench process to phase $\nu^f = 0$ in the PT-symmetry-broken region. The two kinds of Loschmidt rates, DTOPs, Fisher zeros and fixed points are calculated and presented in Fig. S1. We can clearly observe the distinct behaviors of the physical quantities under the two types of bases. The self-normal and biorthogonal Loschmidt rates do not encounter any singularities, as shown in Figs. S1a and S1e. Additionally, the values of DTOPs remain without any jumps in Figs. S1b and S1f. The lines of Fisher zeros also do not cross the real axis (see Figs. S1c and S1g). Meanwhile, there are no fixed points in the dynamics (see Figs. S1d and S1h). These behaviors all indicate the absence of DQPTs in the PT-symmetry-broken region.

S3. MORE DETAILED INFORMATION OF THE EXPERIMENT

In the experiment to observe different types of DQPTs, the initial state is chosen as the pure state $|\psi_{k,-}^{R,i}\rangle = 0.7606\,|H\rangle + 0.6492i\,|V\rangle$. The time evolution in each k-sector is governed by $\hat{U}_k^f$. The density matrix of the evolved state can be rewritten as

$$\rho(k,t) = \left|\Psi_k^R(t)\rangle\langle\Psi_k^R(t)\right| = \frac{1}{2}\sum_{j=0}^{3}\sum_{x_1,x_2} e^{-ik(x_1-x_2)}\,\langle\psi_{x_2}(t)|\,\hat{\sigma}_j\,|\psi_{x_1}(t)\rangle\,\hat{\sigma}_j. \tag{S15}$$

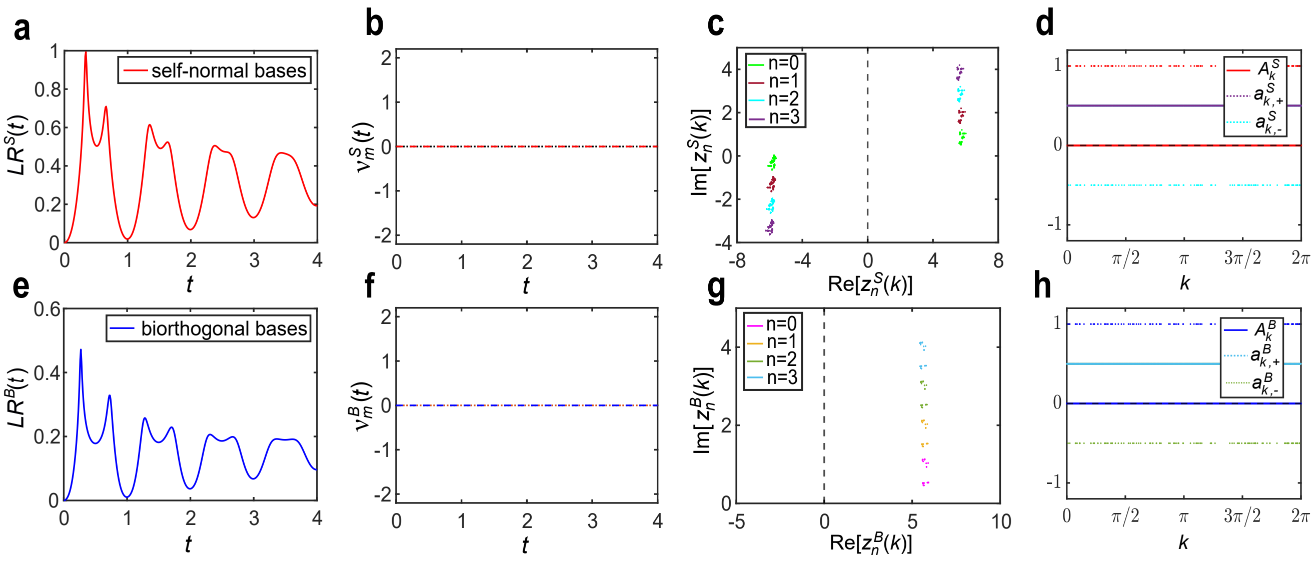

FIG. S1. **Self-normal and biorthogonal DQPTs during a quench between distinct topological phases in the PT-symmetry-broken region.** The Loschmidt rate of $LR(t)$ as a function of time in non-unitary QWs with self-normal bases **a** and biorthogonal bases **e**, respectively. DTOPs of $\nu_m(t)$ as a function of time in non-unitary QWs with self-normal bases **b** and biorthogonal bases **f**, respectively. **c** Real and imaginary parts of the lines of self-normal Fisher zeros $z^S_{n,k}$ with $n=0$ (green), $n=1$ (darkred), $n=2$ (cyan) and $n=3$ (purple). **g** Real and imaginary parts of the lines of biorthogonal Fisher zeros $z^B_{n,k}$ with $n=0$ (magenta), $n=1$ (goldenrod), $n=2$ (darkgreen), $n=3$ (deepskyblue). **d** $A^S_k = a^S_{k,+} - a^S_{k,-}$ as a function of k with $a^S_{k+}=0$ (cyan) and $a^S_{k,-}=0$ (purple). **h** $A^B_k = a^B_{k,+} - a^B_{k,-}$ as a function of k with $a^B_{k,+}=0$ (deepskyblue) and $a^B_{k,-}=0$ (darkgreen). In all panels, the loss parameter is $l=0.5$. The initial state of the QWs is $|x=0\rangle \otimes |\psi^i_-\rangle$ of $\hat{U}^i$ with the coin parameters $(\theta^i_1=\pi/4, \theta^i_2=-\pi/2)$ and the QWs are governed by the final Floquet operator $(\theta^f_1=(\pi-\xi)/2, \theta^f_2=\pi/2)$. All quantities are unitless.

Here $\hat{\sigma}_j$ $(j=0,x,y,z)$ denotes the Pauli matrix, and $|\psi_x\rangle$ represents the coin state at site x and time t. In our experiment, the evolved density matrix $\rho(k,t)$ is reconstructed by performing measurements in position space to extract the matrix elements $\langle\psi_{x_2}(t)|\,\hat{\sigma}_j\,|\psi_{x_1}(t)\rangle$.

For the case $x_1=x_2=x$, the matrix elements can be obtained by performing polarization analysis at each position by performing the projective measurement to the basis states of $\{|H\rangle, |V\rangle, |L\rangle=(|H\rangle - i|V\rangle)/\sqrt{2}, |D\rangle=(|H\rangle+|V\rangle)/\sqrt{2}\}$. This is achieved by applying the combination of a half-wave plate (HWP), a quarter-wave plate (QWP), and a polarizing beam splitter (PBS). By denoting the measured probabilities of photons in these basis states as $P_{\mathrm{H}}(x,t)$, $P_{\mathrm{V}}(x,t)$, $P_{\mathrm{L}}(x,t)$ and $P_{\mathrm{D}}(x,t)$, the matrix elements are given by

$$\begin{aligned}
\langle\psi_x(t)|\,\sigma_0\,|\psi_x(t)\rangle &= P_{\mathrm{H}}(x,t)+P_{\mathrm{V}}(x,t),\\
\langle\psi_x(t)|\,\sigma_x\,|\psi_x(t)\rangle &= 2P_{\mathrm{D}}(x,t)-P_{\mathrm{H}}(x,t)-P_{\mathrm{V}}(x,t),\\
\langle\psi_x(t)|\,\sigma_y\,|\psi_x(t)\rangle &= -2P_{\mathrm{L}}(x,t)+P_{\mathrm{H}}(x,t)+P_{\mathrm{V}}(x,t),\\
\langle\psi_x(t)|\,\sigma_z\,|\psi_x(t)\rangle &= P_{\mathrm{H}}(x,t)-P_{\mathrm{V}}(x,t).
\end{aligned} \tag{S16}$$

For the case $x_1 \neq x_2$, we need to measure

$$\begin{aligned}
\langle\psi_{x_2}(t)|\,\sigma_0\,|\psi_{x_1}(t)\rangle &= a^*_{x_2}(t)a_{x_1}(t)+b^*_{x_2}(t)b_{x_1}(t),\\
\langle\psi_{x_2}(t)|\,\sigma_x\,|\psi_{x_1}(t)\rangle &= a^*_{x_2}(t)b_{x_1}(t)+b^*_{x_2}(t)a_{x_1}(t),\\
\langle\psi_{x_2}(t)|\,\sigma_y\,|\psi_{x_1}(t)\rangle &= -ia^*_{x_2}(t)b_{x_1}(t)+ib^*_{x_2}(t)a_{x_1}(t),\\
\langle\psi_{x_2}(t)|\,\sigma_z\,|\psi_{x_1}(t)\rangle &= a^*_{x_2}(t)a_{x_1}(t)-b^*_{x_2}(t)b_{x_1}(t),
\end{aligned} \tag{S17}$$

where we set the coin state as $|\psi_x(t)\rangle = \left[a_x(t), b_x(t)\right]^{\mathrm{T}}$. Instead of performing projective measurements, we employ interference-based measurements. As illustrated in Fig. 6 of the main text, photons from different spatial modes x_1 and x_2 are combined into the same spatial mode via two HWPs (H_1 and H_2) and beam displacers (BDs). The

polarization state of the resulting photons is then prepared in one of the following four states:

$$\begin{aligned}
&|\phi_1\rangle_{\mathrm{c}} \propto \left[a_{x_1}(t), a_{x_2}(t)\right]^{\mathrm{T}} \text{ when } \mathrm{H}_1 \text{ is at } 0, \mathrm{H}_2 \text{ is at } 45^\circ, \\
&|\phi_2\rangle_{\mathrm{c}} \propto \left[b_{x_1}(t), -b_{x_2}(t)\right]^{\mathrm{T}} \text{ when } \mathrm{H}_1 \text{ is at } 45^\circ, \mathrm{H}_2 \text{ is at } 0 \\
&|\phi_3\rangle_{\mathrm{c}} \propto \left[b_{x_1}(t), a_{x_2}(t)\right]^{\mathrm{T}} \text{ when } \mathrm{H}_1 \text{ is at } 45^\circ, \mathrm{H}_2 \text{ is at } 45^\circ, \\
&|\phi_4\rangle_{\mathrm{c}} \propto \left[a_{x_1}(t), b_{x_2}(t)\right]^{\mathrm{T}} \text{ when removing } \mathrm{H}_1 \text{ and } \mathrm{H}_2.
\end{aligned} \tag{S18}$$

We then perform a projective measurement $\{|L\rangle\langle L|, |D\rangle\langle D|\}$ using a combination of a QWP, a HWP, and a PBS to obtain the photon probabilities in the $\{|L\rangle, |D\rangle\}$ basis. Depending on the polarization state $|\phi_j\rangle_{\mathrm{c}}$ ($j = 1, 2, 3, 4$) of the photons prior to the projective measurement, the corresponding measured probabilities are denoted as $P_L^j(x_1, x_2, t)$ and $P_D^j(x_1, x_2, t)$, respectively.

We are then able to calculate both the real and imaginary parts of $\langle\psi_{x_2}(t)|\sigma_j|\psi_{x_1}(t)\rangle$ ($j = 0, x, y, z$) through

$$\begin{aligned}
\mathrm{Re}\left[\langle\psi_{x_2}(t)|\sigma_0|\psi_{x_1}(t)\rangle\right] &= P_{\mathrm{D}}^1(x_1,x_2,t) - P_{\mathrm{D}}^2(x_1,x_2,t) - \frac{P_{\mathrm{H}}(x_1,t)+P_{\mathrm{H}}(x_2,t)-P_{\mathrm{V}}(x_1,t)-P_{\mathrm{V}}(x_2,t)}{2}, \\
\mathrm{Im}\left[\langle\psi_{x_2}(t)|\sigma_0|\psi_{x_1}(t)\rangle\right] &= P_{\mathrm{L}}^1(x_1,x_2,t) - P_{\mathrm{L}}^2(x_1,x_2,t) - \frac{P_{\mathrm{H}}(x_1,t)+P_{\mathrm{H}}(x_2,t)-P_{\mathrm{V}}(x_1,t)-P_{\mathrm{V}}(x_2,t)}{2}, \\
\mathrm{Re}\left[\langle\psi_{x_2}(t)|\sigma_x|\psi_{x_1}(t)\rangle\right] &= P_{\mathrm{D}}^3(x_1,x_2,t) + P_{\mathrm{D}}^4(x_1,x_2,t) - \frac{P_{\mathrm{V}}(x_1,t)+P_{\mathrm{H}}(x_2,t)+P_{\mathrm{H}}(x_1,t)+P_{\mathrm{V}}(x_2,t)}{2}, \\
\mathrm{Im}\left[\langle\psi_{x_2}(t)|\sigma_x|\psi_{x_1}(t)\rangle\right] &= P_{\mathrm{L}}^3(x_1,x_2,t) + P_{\mathrm{L}}^4(x_1,x_2,t) - \frac{P_{\mathrm{V}}(x_1,t)+P_{\mathrm{H}}(x_2,t)+P_{\mathrm{H}}(x_1,t)+P_{\mathrm{V}}(x_2,t)}{2}, \\
\mathrm{Re}\left[\langle\psi_{x_2}(t)|\sigma_y|\psi_{x_1}(t)\rangle\right] &= P_{\mathrm{L}}^3(x_1,x_2,t) - P_{\mathrm{L}}^4(x_1,x_2,t) - \frac{P_{\mathrm{V}}(x_1,t)+P_{\mathrm{H}}(x_2,t)-P_{\mathrm{H}}(x_1,t)-P_{\mathrm{V}}(x_2,t)}{2}, \\
\mathrm{Im}\left[\langle\psi_{x_2}(t)|\sigma_y|\psi_{x_1}(t)\rangle\right] &= P_{\mathrm{D}}^4(x_1,x_2,t) - P_{\mathrm{D}}^3(x_1,x_2,t) + \frac{P_{\mathrm{V}}(x_1,t)+P_{\mathrm{H}}(x_2,t)-P_{\mathrm{H}}(x_1,t)-P_{\mathrm{V}}(x_2,t)}{2}, \\
\mathrm{Re}\left[\langle\psi_{x_2}(t)|\sigma_z|\psi_{x_1}(t)\rangle\right] &= P_{\mathrm{D}}^1(x_1,x_2,t) + P_{\mathrm{D}}^2(x_1,x_2,t) - \frac{P_{\mathrm{H}}(x_1,t)+P_{\mathrm{H}}(x_2,t)+P_{\mathrm{V}}(x_1,t)+P_{\mathrm{V}}(x_2,t)}{2}, \\
\mathrm{Im}\left[\langle\psi_{x_2}(t)|\sigma_z|\psi_{x_1}(t)\rangle\right] &= P_{\mathrm{L}}^1(x_1,x_2,t) + P_{\mathrm{L}}^2(x_1,x_2,t) - \frac{P_{\mathrm{H}}(x_1,t)+P_{\mathrm{H}}(x_2,t)+P_{\mathrm{V}}(x_1,t)+P_{\mathrm{V}}(x_2,t)}{2},
\end{aligned} \tag{S19}$$

from which we construct all the corresponding matrix elements.

[1] Wang, K. K. et al. Observation of emergent momentum-time skyrmions in parity-time-symmetric non-unitary quench dynamics. *Nature Communications* **10**, 2293 (2019).

[2] Qiu, X. Z. et al. Fixed points and dynamic topological phenomena in a parity-time-symmetric quantum quench. *iScience* **20**, 392-401 (2019).

[3] Wang, K. K. et al. Simulating dynamic quantum phase transitions in photonic quantum walks. *Physical Review Letters* **122**, 020501 (2019).
